# The Influence of Zn and Ca Addition on the Microstructure, Mechanical Properties, Cytocompatibility, and Electrochemical Behavior of WE43 Alloy Intended for Orthopedic Applications

**DOI:** 10.3390/medicina61071271

**Published:** 2025-07-14

**Authors:** Mircea Cătălin Ivănescu, Corneliu Munteanu, Ramona Cimpoeșu, Maria Daniela Vlad, Bogdan Istrate, Fabian Cezar Lupu, Eusebiu Viorel Șindilar, Alexandru Vlasa, Cristinel Ionel Stan, Maria Larisa Ivănescu, Georgeta Zegan

**Affiliations:** 1 Faculty of Dental Medicine, “Grigore T. Popa” University of Medicine and Pharmacy, 16 University Street, 700115 Iasi, Romania; mircea-catalin.ivanescu@umfiasi.ro (M.C.I.); georgeta.zegan@umfiasi.ro (G.Z.); 2 Faculty of Mechanical Engineering, “Gheorghe Asachi” Technical University of Iasi, 43 Dimitrie, Mangeron Blvd, 700050 Iasi, Romania; corneliu.munteanu@academic.tuiasi.ro (C.M.); bogdan.istrate@academic.tuiasi.ro (B.I.); fabian-cezar.lupu@academic.tuiasi.ro (F.C.L.); 3 Faculty of Materials Science and Engineering, “Gheorghe Asachi” Technical University of Iasi, 41 Dimitrie, Mangeron Blvd, 700050 Iasi, Romania; ramona.cimpoesu@academic.tuiasi.ro; 4 TRANSCEND Research Centre, Regional Institute of Oncology, Str. G-ral Henri Mathias Berthelot 2-4, 700483 Iasi, Romania; 5 Faculty of Medical Bioengineering, “Grigore T. Popa” University of Medicine and Pharmacy, 9-13 Kogălniceanu Str, 700454 Iasi, Romania; 6 Faculty of Veterinary Medicine, Iasi University of Life Sciences, Ion Ionescu de la Brad, 700490 Iasi, Romania; eusebiu.sindilar@iuls.ro; 7 Faculty of Dental Medicine, George Emil Palade University of Medicine, Pharmacy, Science, and Technology, 540139 Târgu-Mureș, Romania; alexandru.vlasa@umfst.ro; 8 Faculty of Medicine, “Grigore T. Popa” University of Medicine and Pharmacy, 16 University Street, 700115 Iasi, Romania; cristinel.stan@umfiasi.ro

**Keywords:** Mg alloys, WE43 alloy, biodegradable implants, cytocompatibility

## Abstract

*Background and Objectives*: Magnesium (Mg)-based materials, such as the WE43 alloy, show potential in biomedical applications owing to their advantageous mechanical properties and biodegradability; however, their quick corrosion rate and hydrogen release restrict their general clinical utilization. This study aimed to develop a novel Mg-Zn-Ca alloy system based on WE43 alloy, evaluating the influence of Zn and Ca additions on microstructure, mechanical properties, cytocompatibility, and electrochemical behavior for potential use in biodegradable orthopedic applications. *Materials and Methods*: The WE43-Zn-Ca alloy system was developed by alloying standard WE43 (Mg–Y–Zr–RE) with 1.5% Zn and Ca concentrations of 0.2% (WE43_0.2Ca alloy) and 0.3% (WE43_0.3Ca alloy). Microstructural analysis was performed utilizing scanning electron microscopy (SEM) in conjunction with energy-dispersive X-ray spectroscopy (EDS), while the chemical composition was validated through optical emission spectroscopy and X-ray diffraction (XRD). Mechanical properties were assessed through tribological tests. Electrochemical corrosion behavior was evaluated using potentiodynamic polarization in a 3.5% NaCl solution. Cytocompatibility was assessed in vitro on MG63 cells using cell viability assays (MTT). *Results*: Alloys WE43_0.2Ca and WE43_0.3Ca exhibited refined, homogeneous microstructures with grain sizes between 70 and 100 µm, without significant structural defects. Mechanical testing indicated reduced stiffness and an elastic modulus similar to human bone (19.2–20.3 GPa), lowering the risk of stress shielding. Cytocompatibility tests confirmed non-cytotoxic behavior for alloys WE43_0.2Ca and WE43_0.3Ca, with increased cell viability and unaffected cellular morphology. *Conclusions*: The study validates the potential of Mg-Zn-Ca alloys (especially WE43_0.3Ca) as biodegradable biomaterials for orthopedic implants due to their favorable combination of mechanical properties, corrosion resistance, and cytocompatibility. The optimization of these alloys contributed to obtaining an improved microstructure with a reduced degradation rate and a non-cytotoxic in vitro outcome, which supports efficient bone tissue regeneration and its integration into the body for complex biomedical applications.

## 1. Introduction

Mg-based materials and their alloys have attracted considerable interest in advanced engineering and biomedical applications due to their low density, favorable mechanical properties, and biodegradability [1]. Mg-based alloys exhibit good specific mechanical properties but have low corrosion resistance and moderate absolute properties, which limits their widespread applicability [2,3,4]. By modifying their chemical composition and applying compatible surface treatments, Mg alloys—including WE43 (Mg–Y–Zr–RE cast ingot)—can be optimized to improve degradation rate and bone integration, while also reducing adverse effects such as molecular hydrogen formation during corrosion [5,6,7,8,9,10,11].

WE43 alloy is recognized as a promising material for biomedical applications due to its superior mechanical properties and biodegradability. However, its clinical use is limited by its accelerated degradation rate and hydrogen release during corrosion, which necessitates advanced surface protection strategies and compositional optimization to expand its use in orthopedic and cardiovascular implants [12,13]. WE43 contains rare earth (RE) elements, which contribute to the formation of a protective oxide layer, thereby improving corrosion resistance and enabling controlled degradation in complex physiological environments [14,15].

The microstructure and properties of WE43 alloys can be adjusted through modern processing techniques, including metal–gas eutectic solidification, pressure casting, infiltration, powder metallurgy, Selective Laser Melting (SLM), Directed Metal Deposition (DMD), and Powder Bed Fusion with Laser Beam/Metal (PBF-LB/M) [16]. Thermal treatments allow for the refinement of microstructure, mechanical and corrosion property enhancement, and the development of efficient corrosion-protective layers due to the formation of nanoscale precipitates, which significantly improve alloy strength [17,18].

The careful selection of alloying elements and surface treatments can enhance corrosion resistance, and when the degradation rate is controlled, certain Mg alloys can be used as bioresorbable implant materials. For example, the simultaneous addition of zinc (Zn) and RE elements promotes the formation of a long-period stacking ordered (LPSO) structure, significantly enhancing mechanical strength [2]. Thermal stability is improved by RE elements such as yttrium (Y) and gadolinium (Gd), which form intermetallic phases that limit corrosion propagation through galvanic mechanisms [19,20,21,22,23]. Additionally, zirconium (Zr) improves mechanical strength and fatigue behavior by refining grain structure [24].

The addition of calcium (Ca) to magnesium-based alloys in amounts between 0.4% and 1% facilitates grain refinement, augments mechanical strength, and diminishes corrosion rates by promoting the creation of a protective Mg(OH)_2_ layer [25]. However, excessive Ca content (>1%) can lead to the formation of Mg_2_Ca intermetallic phases, which increase the likelihood of localized corrosion and embrittlement, thus limiting biomedical usability [25,26]. Cha et al. (2013) [27] observed that adding Zn to Mg-Ca alloys modified the corrosion potentials of the constituent phases (Mg and Mg_2_Ca), thereby preventing galvanic coupling and achieving a corrosion rate comparable to that of high-purity Mg. In Mg-Zn-Ca alloys, Ca may play a similar role to RE elements in influencing recrystallized texture evolution [28]. The addition of Ca in concentrations from 0.5% to 4% by weight significantly improves both mechanical strength and corrosion resistance [20,24,27,29,30].

Zn-based alloys exhibit improved mechanical properties and osteoconductivity, but excessive Zn release during degradation can be toxic at high concentrations [31]. Reducing Zn content and balancing it with Ca led to the development of a high-strength, low-corrosion alloy: Mg-0.45Zn-0.45Ca (wt.%) (ZX00) [32]. At approximately 1.5 wt.%, Zn strengthens solid solutions and reduces corrosion by forming stable intermetallic phases that support the development of a Mg(OH)_2_ protective layer [4,33,34,35,36]. Kumar and Pandey (2020) [37] developed a powder-based biomaterial for biomedical implantation by combining Mg with Nb, Zn, and Ca.

In vitro studies have shown that the main challenge in Mg-based biomaterials lies in their high corrosion rate in physiological environments, which rapidly generates byproducts such as Mg^2+^ ions, hydroxides (OH^−^), and hydrogen gas (H_2_). These byproducts may alkalinize the local environment and compromise mechanical integrity before healing is complete, making surface modification or alloying with biocompatible elements necessary to improve performance [15,38]. Mg-Ca alloys offer a balance between biocompatibility and biodegradability, although the degradation rate varies significantly with Ca content. In vitro studies have demonstrated that higher Ca concentrations increase hydrogen evolution and pH, accelerating degradation, while lower-Ca alloys, such as Mg-0.5Ca, exhibit improved corrosion resistance and more controlled degradation, making them more suitable for biodegradable implants [39]. Soluble Mg^2+^ and Ca^2+^ ions, along with insoluble corrosion products, affect cell viability and myotube formation, with a higher Ca/Mg ratio favoring myotube development [40].

The in vitro performance of WE43 alloy demonstrated better corrosion resistance than pure Mg, with a lower degradation rate and more efficient formation of a stable surface protective layer [41]. The in vitro behavior of Mg-Y-Zn-Zr-Ca alloys showed an initial rapid dissolution, followed by stabilization due to the formation of a protective hydroxide and calcium compound layer, which limited the release of metal ions. The presence of Ca supported the formation of a bioactive protective structure, facilitating osteogenic cell interaction and promoting osteointegration [42].

In this study, the WE43-Zn-xCa alloy system was developed through alloying the standard WE43(Mg–Y–Zr–RE) cast alloy used as the base material with 1.5% Zn and Ca concentrations of 0.2% and 0.3% in order to improve the mechanical properties and corrosion behavior of the WE43 alloy. The objective was to develop a new Mg-based alloy system, WE43-Zn-xCa, and to assess the influence of Zn and Ca additions on microstructural analysis, mechanical properties, cytocompatibility, and electrochemical behavior for potential use in biodegradable orthopedic applications.

## 2. Materials and Methods

### 2.1. Alloy Fabrication

The WE43–1.5Zn–xCa system was designed by alloying 1.5% Zn and xCa (x = 0.2% and 0.3%) into the standardized WE43 alloy. The two resulting alloys were used in our study as experimental alloys; WE43-1.5Zn-0.2Ca was named WE43_0.2Ca and WE43-1.5Zn-0.3Ca was named WE43_0.3Ca. The system was fabricated using an induction melting Rotocast furnace in ceramic crucibles under an argon atmosphere to prevent ignition and oxidation of the alloy. The casting batch consisted of high-purity master alloys (99.995%), Mg–15%Ca and Mg–20%Zn, and the WE43 standardized cast ingot alloy, which contains the other elements from the Mg–Y–Zr–RE system. Post-casting, a homogenization heat treatment was performed under the same controlled argon atmosphere at 420 °C for 12 h, followed by slow cooling.

### 2.2. Characterization of Microstructural, Chemical Composition, and Mechanical Properties 

After casting the alloy, a heat treatment was performed, followed by sectioning of the ingots using a Metacut 302, Metkon, Bursa, Turkey machine. The samples were then embedded in resin using the Metkon Ecopress 52, Metkon, Bursa, Turkey device. Preliminary sample preparation involved mechanical grinding with abrasive papers of various grit sizes until minimal surface roughness was achieved, using the Forcipol 202, Metkon, Bursa, Turkey polisher. This was followed by metallographic chemical etching using specific etchants.

For sample examination via SEM, and EDS, a Thermo Scientific Quattro C microscope, Brno, Czech Republic was used. This technique enabled comprehensive characterization of the Mg alloy, serving a dual purpose: first, a microstructural and defect analysis was carried out from a materials engineering perspective, and second, chemical composition and the distribution of biocompatible elements within the WE43-Zn-xCa alloy system were evaluated from a biomedical standpoint. The metal samples underwent microstructural examination through scanning electron microscopy (SEM) at magnifications of 100×, 500×, 5000×, and 10,000×. This facilitated a comprehensive analysis of grain size, shape, and distribution. SEM was utilized to examine the surface and morphology at both the micrometer and nanometer scale, offering high-resolution imaging and analytical flexibility. SEM enabled the observation of surface texture and topography, which is essential for characterizing thin films, coatings, or chemically treated surfaces. It also facilitated the identification of cracks, voids, inclusions, or other defects that may affect the material’s performance.

The identification of intermetallic phases in the alloy was performed using X-ray diffraction (XRD) analysis with an X’Pert PRO MPD, Panalytical, Almelo, The Netherlands, diffractometer equipped with a Cu X-ray tube (Kα = 1.54051 Å): 2 theta range was 100–900, step size was 0.13, time per step was 51 s, and scan speed was 0.0656510/s. The Panalytical X’Pert PRO X-ray diffractometer was configured for phase and compound analysis, as well as analyzing the diffraction of thin films in terms of crystallinity, semi-quantitative chemical composition, texture, and internal stresses. To achieve optimal performance, the X-ray tube was configured at 40 kV and 45 mA.

The chemical composition was determined using the Foundry Master Smart, (Wetzlar and Mannheim, Germany) optical emission spectrometer.

Surface properties were assessed using the UMTR 2M-CTR tribometer, version 1.122.245. The tribological behavior of the alloys was analyzed with the UMT Test Viewer software, CP4–2.16.93+, and the XRD graphs were processed using OriginPro 8.5. The test samples were prepared in the form of disks with a diameter of 50 mm and a thickness of 2.5–3 mm. Hardness and longitudinal elastic modulus were measured using the microindentation method with the UMTR 2M-CTR tribometer. The tribometer features a precise indenter that produces a consistent and regulated scratch trace on the surface of the material. The experiments were conducted applying a peak vertical force of 10 N, with the stage traversing a distance of 10 mm within a duration of 60 s, at a velocity of 0.167 mm/s. Scratch tests were conducted using an NVIDIA-type blade indenter with a tip radius of 0.4 mm. For indentation testing, a Rockwell-type diamond indenter with a tip angle of 120° and a spherical tip radius of 200 μm was used, applying a maximum force of 10 N.

### 2.3. Characterization of Electrochemical Corrosion 

The OrigaFlex potentiostat was used for electrochemical corrosion testing and for characterizing the corroded surfaces, while the OrigaMaster5 software (v 2.5.0.5) was employed to interpret the experimental results.

Measurements were conducted using a three-electrode corrosion cell in a potentiodynamic manner. Teflon washers were utilized to secure the samples—flat disks measuring 10 mm in diameter and 1 mm in thickness—within the working electrode, resulting in flat circular surfaces of up to 0.8 cm^2^. The exposed surface area in this instance was measured at S = 0.503 cm^2^. A saturated calomel electrode was utilized as the reference electrode, while a platinum wire electrode functioned as the counter electrode. A 3.5% sodium chloride (NaCl) solution served as the electrolyte. The measurements were performed at 23 °C, and the electrolyte was naturally aerated.

Tafel extrapolation, performed using the OrigaMaster5 software, allowed for the determination of the corrosion potential at zero current (E_0_ ≡ E(I = 0)), the anodic (ba) and cathodic (bc) Tafel slopes, the polarization resistance (Rp), the corrosion current density (Jcor), and the corresponding corrosion rate (vcor). The potential range of linear anodic polarization was from −300 mV to +300 mV relative to the open-circuit potential (OCP) at a scan rate of 1 mV/s. The impedance spectra were recorded over a frequency range of 100 kHz to 10 mHz, with a potential amplitude of 10 mV. The experimental data were subsequently interpreted through fitting them to equivalent circuit models appropriate for characterizing corrosion behavior. Electrochemically corroded and freshly ground samples were examined using a Vega Tescan LMH II scanning electron microscope (TESCAN ORSAY HOLDING a.s., Brno, Czech Republic, 30 kV, SE detector, high-pressure mode). An EDX QUANTAX QX2 EDS detector, connected to the Bruker/Roentec Co., electron microscope (Berlin, Germany), was used to evaluate the chemical composition across different surface regions of both corroded and untreated alloy samples.

### 2.4. Cytocompatibility Assay

#### 2.4.1. Cell Culture

For this study, the MG-63 cell line (i.e., human osteosarcoma osteoblast commercial cell line) was selected. Cells were thawed and cultured in MEM (Minimal Eagle medium; Sigma-Aldrich, St. Louis, MO, USA) culture medium supplemented with 10% fetal bovine serum (FBS; Sigma-Aldrich, USA) and 1% antibiotic (penicillin–streptomycin; Sigma-Aldrich, USA) in 75 cm^2^ culture flasks, in standard conditions (i.e., at 37 °C and 5% CO_2_), until confluence. Then, the culture media was removed, the adherent cells were washed with phosphate-buffered saline solution (PBS), detached with trypsin–EDTA, and centrifuged, and the cell pellet was suspended in complete culture medium in order to obtain the working suspension from which the 48-well cell culture plates were seeded at a density of 104 cells/well for cytocompatibility evaluation at 1 and 3 days.

#### 2.4.2. Alloy Samples Preparation

The alloy samples with a well-defined mass were cleaned by sonication with ethanol and then exposed to UV for 30 min on each side. Subsequently, they were immersed for 48 h in completely sterile MEM culture media (maintained in an environment with 5% CO_2_, relative humidity > 95%, and 37 °C) at an extraction ratio of 0.1 g/mL (according to ISO-10993-12 and ISO-10993-5 standards [43,44]), to use several dilutions of the resulting extracts (i.e., dilutions of 10%, 20%, 40%, 60%, 80%, 100% per well) for the cytotoxicity study. Wells containing only cultured cells (i.e., control wells), without any extract, were used as the negative control.

#### 2.4.3. Cell Viability

Cell viability was tested using MTT (as was published by Vlad et al. [45,46,47], a tetrazolium salt (3-[4,5dimethylthiazol-2yl] 2,5 diphenyltetrazolium bromide) solution, which is able to penetrate the viable cells’ membrane and, through the action of the mitochondrial enzymes, is converted to intracellular, blue-colored, formazan crystals. The color intensity of the formazan crystals solubilized in DMSO, which is directly related to the number of viable cells, was measured by using a spectrophotometer (FilterMax F5 Multimode Microplate Reader; Molecular Devices, Munchen, Germany) at a wavelength of 570 nm. The test was performed in triplicate. The cell viability obtained after 1 and 3 days was expressed as a percentage in relation to the control wells, according to the following formula: Viability (%) = 100x(Ae/Ac), where Ae represents the absorbance of the studied alloy extracts and Ac is the absorbance of the control wells (i.e., cells cultured only with the culture media, without extract addition).

The one-way ANOVA test was used for statistical analysis of the cell viability results, and the Tukey method was used to compare the data. Statistically significant differences were noted when *p* < 0.05.

#### 2.4.4. Cell Morphology

After coincubation of the MG63 cells with extracts containing 100% of the studied WE43_0.2Ca and WE43_0.3Ca alloys for 3 days (under the above-specified conditions), the cells were washed with HBSS (Hanks’ Balanced Salt solution) without red phenol and subsequently stained with 1:1000 Calcein–AM (C1359, Sigma-Aldrich) solution in HBSS through incubation for 30 min in the dark at 37 °C. After that, cell morphology was evaluated with an inverted fluorescence microscope (Leica DMIL LED, equipped with Leica DFC450C camera and image acquisition software—Leica Application Suite—Version 7.4.1; Leica, Wetzlar, Germany).

## 3. Results

### 3.1. Microstructural Analysis

**Figure 1:**
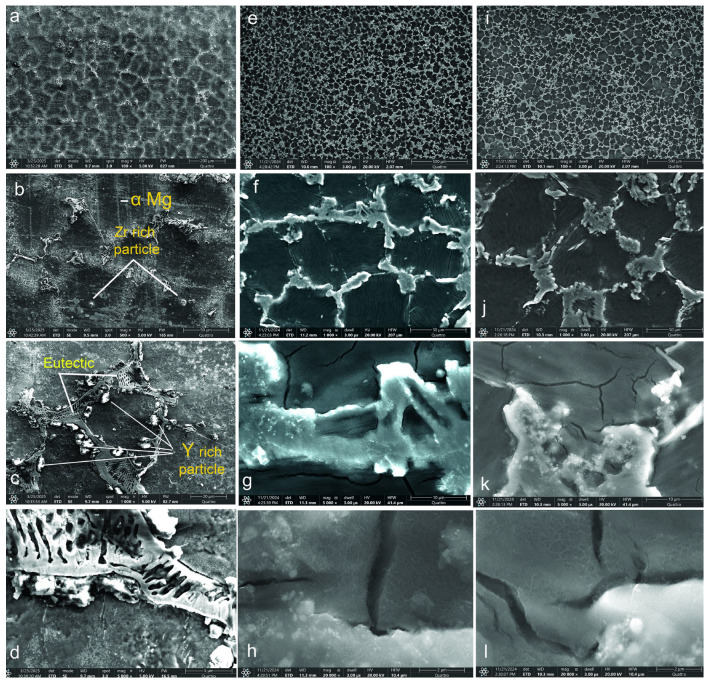
SEM images of the alloys: WE43 (**a**–**d**); WE43_0.2Ca (**e**–**h**); WE43_0.3Ca (**i**–**l**).

SEM images for the WE43, WE43_0.2Ca, and WE43_0.3Ca alloys are shown in Figure 1.

Microstructural analysis of the WE43 alloy revealed grain sizes ranging from 100 to 130 μm (Figure 1a,b), while alloys WE43_0.2Ca and WE43_0.3Ca (Figure 1e,f and Figure 1i,j, respectively) exhibited reduced grain sizes of between 70 and 100 μm with a well-defined polyhedral morphology. This feature indicates controlled solidification without major crystallization disturbances. At 500× magnification (Figure 1b,f,j), the grain boundaries are clearly visible, suggesting a homogeneous microstructure without segregation or structural irregularities. Uniformly dispersed inclusions in the metallic matrix confirm the presence of secondary phases resulting from the local segregation of alloying elements. These phases enhance mechanical strength and durability through strengthening dispersion. The uniform grain distribution suggests homogeneous nucleation during solidification, contributing to thermal and mechanical stability. A uniform cooling rate and effective control of the solidification process are inferred from the absence of significant grain size variations. Electron microscopy confirmed the absence of segregation, microcracks, porosity, or solidification defects, indicating that the optimized fabrication process produced an alloy with uniform structural characteristics and improved mechanical performance.

The addition of 1.5% Zn and 0.2–0.3% Ca resulted in microstructural refinement through a uniform distribution of α-Mg grains, which is consistent with previous observations on Mg-based alloys alloyed with Ca [48,49]. In the case of the AZ91D alloy, it was reported that adding Ca up to 0.3–0.4 wt.% led to significant grain refinement, with grain size reduced by a quarter; however, further additions of Ca above 1.0 wt.% no longer contributed to additional grain size reduction [49]. Small amounts of calcium (0.4–0.8 wt.%) lead to grain refinement, improved corrosion resistance, and the formation of intermetallic phases, particularly Mg_2_Ca [20].

Additionally, the formation of the Mg_2_Ca intermetallic phase and ternary Ca-Mg-Zn compounds situated at the grain boundaries was facilitated, which showed a lamellar structure. Research in references [50,51] supports these findings for the Ca-Mg-Zn system with various additions. The Mg_2_Ca phase plays a significant role in the formation of a Mg(OH)_2_ layer, serving as a protective barrier against corrosion in physiological environments while also enhancing the matrix via dispersion mechanisms. Lu et al. showed that the biocorrosion rate of the WE43_0.3Ca alloy is closely linked to grain size and the volume fraction of the secondary phase [52].

The WE43 alloy microstructure consists of an α-Mg matrix in which Y and Nd are typically in solid solution, along with multiple precipitate types distributed both at and within grain boundaries [53]. The most common precipitates in WE43 are based on the ternary Mg-Y-Nd system [54]. Rectangular precipitates observed at grain boundaries were identified as Mg_24_Y_5_ [55,56], while fine Mg_41_Nd_5_ phase particles and globular Zr-rich precipitates are also present [56,57].

### 3.2. Chemical Composition: Energy-Dispersive X-Ray Spectroscopy (EDS)

To validate the chemical composition and confirm the presence of the alloying elements Zn and Ca in the WE43_0.2Ca and WE43_0.3Ca alloys, optical emission spectrometry (OES) analysis was utilized. This method facilitated swift and precise identification of the chemical makeup, encompassing both primary and trace elements. The findings derived from the spectrometric analysis are detailed in Table 1, illustrating the chemical composition of the WE43, WE43_0.2Ca, and WE43_0.3Ca alloys.

**Figure 2:**
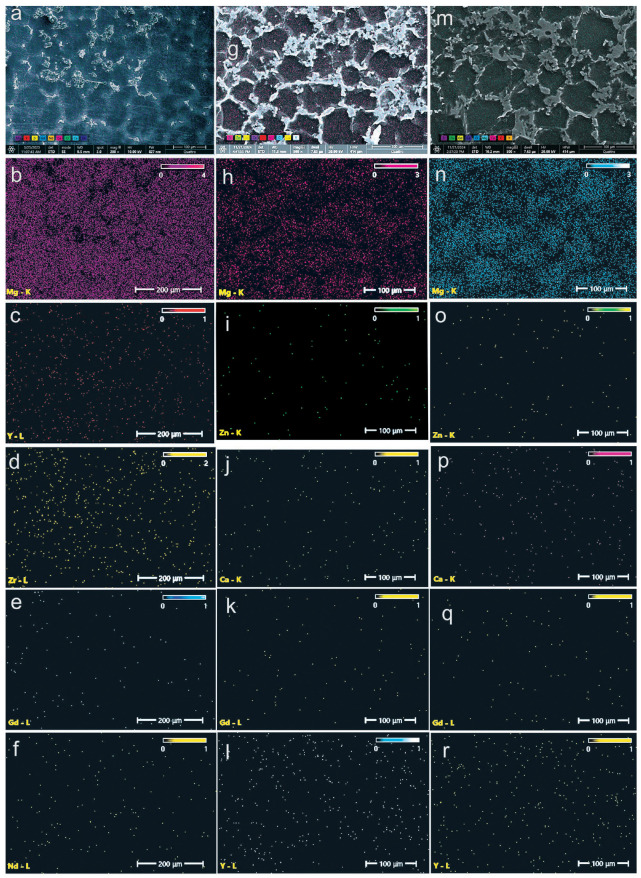
EDS images showing the chemical element distribution on the surface of the alloys WE43 (**a**–**f**), WE43_0.2Ca (**g**–**l**), and WE43_0.3Ca (**m**–**r**).

The combination of SEM and EDS techniques enabled a detailed examination of the material for both the standard WE43 alloy (Figure 2a–f), and the two alloys from the WE43–1.5Zn–xCa system (Figure 2g–r). SEM provided information on the physical structure of the alloy system, while EDS supplied chemical data, leading to a comprehensive understanding of the WE43–1.5Zn–xCa alloy system. The integration of these two methods for analyzing structural morphology and surface chemical composition facilitated the mapping of the distribution of alloying elements within the microstructure. This allowed for the visualization of the spatial distribution of elements in both the base material, WE43, and in the two experimentally obtained alloys. Figure 2 illustrates the distribution of elements on the surface of the analyzed alloys.

In the case of the standard WE43 alloy (Figure 2a–f), the elemental distribution of Y, Nd, and Zr is relatively homogeneous, with slight segregation observed along grain boundaries. This behavior is consistent with the formation of intermetallic precipitates characteristic of the Mg–Y–Nd and Mg–Zr systems [53,57].

In contrast, for the experimental alloys WE43_0.2Ca and WE43_0.3Ca, EDS analyses reveal a more homogeneous distribution of the alloying elements Zn and Ca (Figure 2g–r), primarily concentrated at the grain boundaries. This spatial localization indicates the formation of stable secondary phases, such as Mg_2_Ca and ternary Ca–Mg–Zn compounds exhibiting lamellar morphology. The observed distribution confirms the effective incorporation of Zn and Ca into the microstructure and supports the hypothesis that their addition promotes grain refinement and contributes to improved electrochemical behavior through the stabilization of corrosion-resistant intermetallic phases.

### 3.3. Electrochemical Corrosion Resistance Characterization

**Figure 3:**
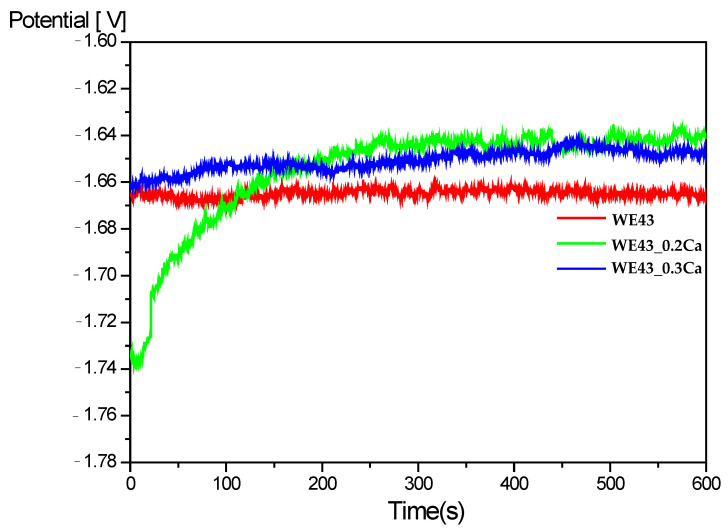
Open circuit potential (OCP) in 3.5% NaCl electrolyte.

The Open Circuit Potential (Eₒc) time dependence for the WE43, WE43_0.2Ca and WE43_0.3Ca samples immersed in a 3.5% NaCl electrolyte is shown in Figure 3 [58]. A rather steep increase in OCP toward more positive values can be observed for both alloyed samples, WE43_0.2Ca (after approximately 200 s) and WE43_0.3Ca, eventually reaching a constant value of around –1.65 V after 600 s. This increase in OCP is likely associated with the formation of a corrosion layer that progressively improves its protective characteristics over time. The stabilization of the OCP after 600 s suggests that electrochemical equilibrium has been reached and that the surface has been uniformly coated by the corrosion product layer.

**Figure 4:**
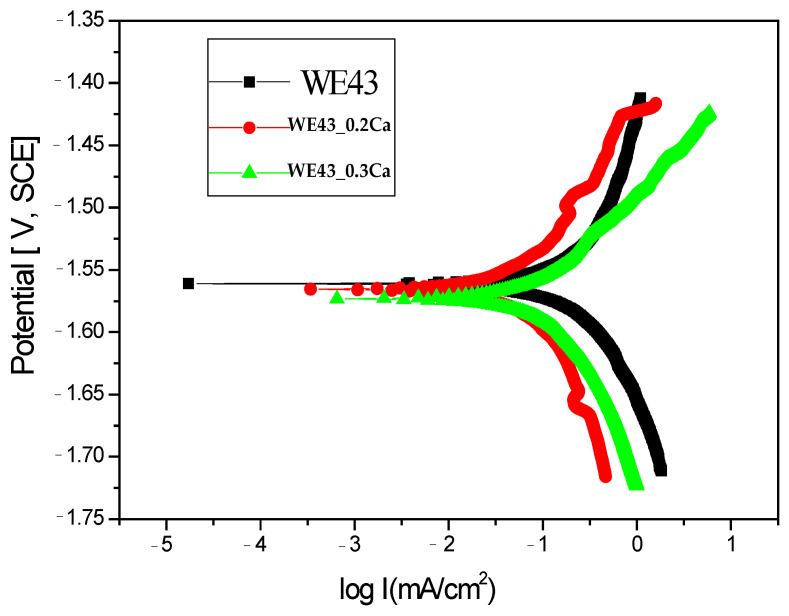
Tafel diagrams of All Samples Tested in 3.5% NaCl.

In order to evaluate the corrosion behavior, the Tafel curves obtained for the three samples are presented in Figure 4. It is well known that samples with more electropositive corrosion potentials exhibit better corrosion resistance. Table 2 presents the key electrochemical parameters obtained for the three samples in the 3.5% NaCl electrolyte.

Electrochemical measurements indicated that the corrosion potentials (E_cor_) for the WE43_0.2Ca and WE43_0.3Ca samples were nearly identical to that of the WE43 sample, exhibiting only slight potential variations. Upon analyzing the corrosion current density values, it is evident that there is an enhancement in the corrosion resistance of the WE43_0.2Ca and WE43_0.3Ca samples. The corrosion current density shows a reduction from 0.19 mA/cm^2^ for the WE43 sample to 0.11 mA/cm^2^ for the WE43_0.3Ca sample. The enhancement observed can be largely credited to the structural uniformity attained in the newly formulated WE43_0.2Ca and WE43_0.3Ca alloys, and it may also be associated with the development of a protective corrosion layer on the second-phase precipitates.

Moreover, the polarization resistance exhibited the most notable enhancement in the scenario using the WE43_0.2Ca sample.

Regarding corrosion rate (Table 2), the WE43_0.3Ca alloy exhibited the lowest value (2.6 mm/Y), while 4.59 mm/Y was observed for the WE43 alloy. This may be attributed to the more intense anodic reaction in WE43, due to a larger active area of the α-Mg matrix being exposed to the corrosive medium. 

**Figure 5:**
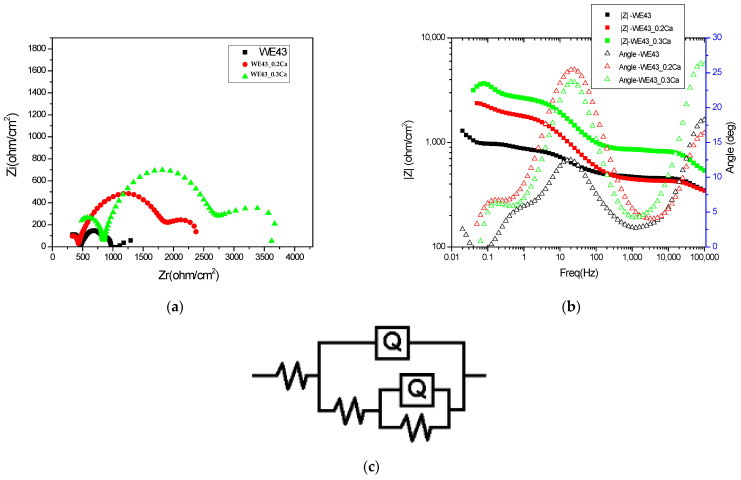
Electrochemical impedance diagrams: (**a**) Nyquist representations; (**b**) Bode representation; (**c**) schematic representation of the equivalent circuit.

The electrochemical impedance spectra in Bode and Nyquist representations, recorded for the three investigated samples and shown in Figure 5a,b, illustrate differences in the charge transfer mechanisms and barrier properties of the formed layers, providing a comparative view of the electrochemical stability of the analyzed systems. A progressive increase in total impedance is observed with the increasing calcium content, suggesting improved electrochemical stability of the surface exposed to the corrosive environment. The Bode plots show a gradual increase in impedance modulus at low frequencies with higher Ca content, indicating enhanced barrier behavior and reduced electrochemical activity at the interface. The presence of two time constants, reflected by two distinct slopes in the phase diagram, suggests the existence of two superimposed layers: an outer porous layer and an inner compact layer. The increase in low-frequency impedance, correlated with a decrease in the capacitive dispersion (Q_2_) and an increase in resistance (R_2_), confirms the reinforcement of the inner protective layer upon the addition of calcium to the alloy’s composition. EIS spectra modeling was performed using the equivalent circuit R(Q(R(QR))) (Figure 5c), which allows for the separation of electrochemical contributions from different regions of the corrosion film. In this model, the solution resistance (R_s_) reflects the ionic conductivity of the electrolyte, while the constant phase element Q_1_ and its associated resistance R_1_ are attributed to the outer porous layer composed of more permeable corrosion products. The inner component Q_2_–R_2_ describes the behavior of the compact inner layer, which is the primary protection against charge transfer at the metal–electrolyte interface. This model enables the identification of how the multilayered structure of the surface film influences barrier properties and electrochemical stability, in correlation with the compositional variability of the alloy.

The approximately constant values of the solution resistance (R_s_) across the three alloys, as shown in Table 3, indicate a stable conductivity of the testing medium (3.5% NaCl), which is not significantly affected by the compositional variations in the alloys. This behavior suggests that the observed differences in impedance response are not influenced by changes in the electrolyte properties, but mainly reflect modifications in the passive layers formed on the sample surfaces. The nearly constant values of the constant phase element Q_1_ indicate that the capacitive properties of the outer porous layer are minimally affected by the variation in Ca content. The stability of Q_1_ also reflects a relatively uniform roughness and porosity in the outer layer across the entire series of investigated samples. The increase in R_1_ values with higher Ca content indicates a reduction in the permeability of the outer porous layer, possibly due to the deposition of additional corrosion products or structural consolidation of this layer. The presence of Ca likely promotes the formation of less soluble compounds that can block the pores of the outer layer and reduce ionic transport through it, thereby contributing to increased diffusion resistance and improved overall barrier performance. Lower Q_2_ values suggest a decrease in interfacial capacitance, most likely associated with reduced defects and reduced porosity in the inner layer. Thus, the addition of Ca promotes the formation of denser films with a more pronounced dielectric character, which contributes to enhanced corrosion resistance, as also reflected by the increased R_2_ values.

### 3.4. Mechanical Properties

**Figure 6:**
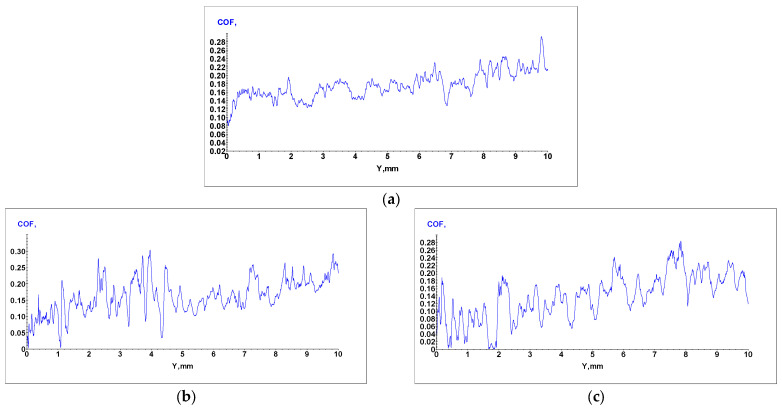
Apparent coefficient of friction (COF) for WE43 (**a**), WE43_0.2Ca (**b**), and WE43_0.3Ca (**c**) alloys.

**Figure 7:**
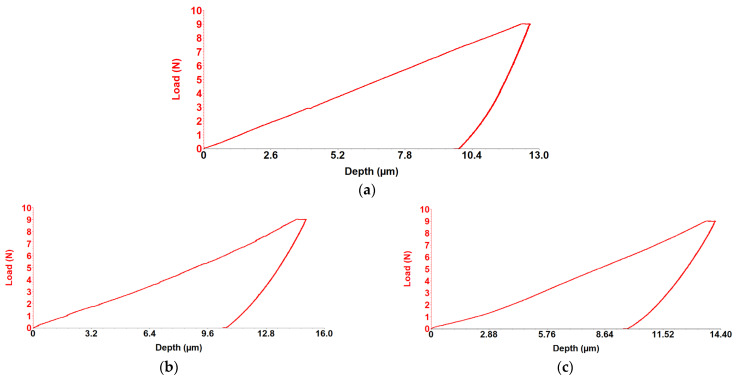
Load–depth curve graph for alloy WE43 (**a**), WE43_0.2Ca (**b**) and WE43_0.3Ca (**c**) alloy.

Sliding Friction Coefficient was obtained using the scratch test technique, and results for the WE43, WE43_0.2Ca, and WE43_0.3Ca alloys are presented in the graphs in Figure 6a–c. The indentation test results for the WE43, WE43_0.2Ca, and WE43_0.3Ca alloys are shown in the graphs in Figure 7a–c.

Following the alloying of WE43 with Zn and Ca, a reduction in stiffness and elastic modulus was observed. The obtained Young’s modulus values were 19.2 GPa for WE43_0.2Ca and 20.3 GPa for WE43_0.3Ca (Table 4)—within the range of human bone (10–30 GPa) [59,60]—an important factor in reducing the risk of stress shielding in biodegradable bone fixation implants. The coefficient of friction was 0.17 for WE43, compared to 0.16 for WE43_0.2Ca and 0.14 for WE43_0.3Ca (Table 4). With penetration depths of 11.2 μm (WE43), 12.6 μm (WE43_0.2Ca), and 11.8 μm (WE43_0.3Ca) (Table 4), these values correlate with Young’s modulus, stiffness, and strength, indicating that WE43_0.2Ca and WE43_0.3Ca are more elastic alloys with a slightly lower wear resistance than WE43. The decreased stiffness, combined with the lower friction coefficient and an elastic modulus closer to that of human bone, qualifies the WE43_0.3Ca alloy for repeated long-term use, with greater abrasion resistance and less localized wear than WE43_0.2Ca, positioning it as a promising biomaterial candidate for future research.

A promising direction for further research involves the development of computational models to build digital equivalents of Mg-based alloys, allowing for faster and more resource-efficient alloy design [2]. Digital tools could facilitate the production of porous Mg structures with high applicability in biomedical engineering. Porous structures produced using the SLM method applied to Mg-Zn-Zr-Ca alloys have demonstrated mechanical properties similar to cortical bone, with an elastic modulus of 42 GPa—close to that of bone—allowing for osteoblast integration and new bone tissue formation [61].

### 3.5. X-Ray Diffraction (XRD)

**Figure 8:**
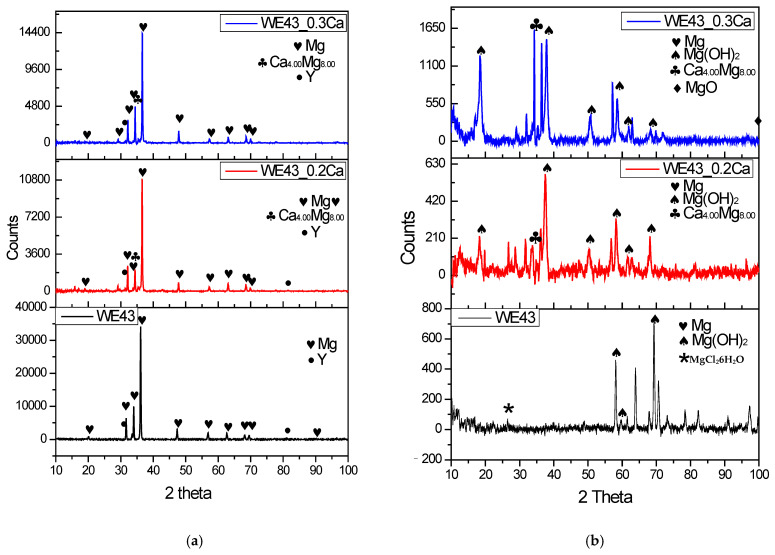
XRD spectra of WE43, WE43_0.2Ca, and WE43_0.3Ca alloys before electrochemical corrosion (**a**) and after electrochemical corrosion (**b**).

The XRD spectra of the WE43, WE43_0.2Ca, and WE43_0.3Ca alloys before electrochemical corrosion are shown in Figure 8a, and the XRD spectra after corrosion are shown in Figure 8b.

Before electrochemical corrosion (Figure 8a), XRD analysis showed that the most intense peak corresponded to the Mg 2.00 crystalline phase at 2θ = 36.2° for WE43, 2θ = 36.54° for WE43_0.2Ca, and 2θ = 36.61° for WE43_0.3Ca, (JCPDS 35-0821). The most intense peak corresponding to Y occurred at 2θ = 32.3 (JCPDS 00-034-1491). The intermetallic Ca_4_Mg_8_ phase was identified in both WE43_0.2Ca and WE43_0.3Ca at 2θ = 34.74° (JCPDS 00-053-0461), consistent with findings in the specialized literature [62,63,64].

After electrochemical corrosion (Figure 8b) XRD revealed the presence of Mg(OH)_2_ with maximum peaks at 2θ = 38.19° and 18.66° for WE43 and WE43_0.2Ca, and at 2θ = 43.6° and 18.3° for WE43_0.3Ca(JCPDS 7-0239), supporting previous findings [64,65]. Severe Mg dissolution is supported by the XRD results showing weak or absent Mg(OH)_2_ peaks in the corroded WE43 sample (Figure 8). The inconsistent intensity of these peaks is likely due to the heterogeneous and constant spalling of corrosion products, which results in an uneven, roughened surface. In contrast, WE43_0.2Ca and WE43_0.3Ca alloys, which displayed lower corrosion rates, formed more compact and adherent Mg(OH)_2_ layers, as confirmed by their respective XRD peaks.XRD analysis performed after corrosion revealed the presence of the MgCl_2_6H_2_O phase (JCPDS 70-2151), with a maximum peak at 2θ = 27.2°, which was considered a product of the interaction between the ions released during the electrochemical process and the saline environment, resulting from secondary precipitation reactions at the exposed surface. Its presence in the WE43 sample suggests a strong local interaction with Cl^−^ ions in the solution, without directly indicating a specific corrosion mechanism or any protective or accelerating influence. In comparison, this phase was not clearly detected in the WE43_0.2Ca and WE43_0.3Ca alloys, which may be attributed to a difference in local chemical equilibrium caused by the microstructural changes induced by Zn and Ca additions.

### 3.6. Cytocompatibility Results 

**Figure 9:**
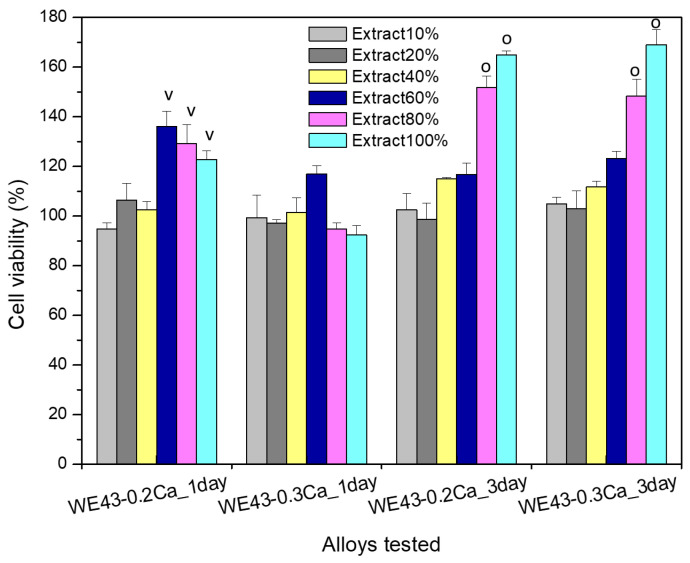
Results of the MTT assay for the study of the cell viability of MG63 osteoblasts coincubated, for 1 and 3 days with WE43_0.2Ca and WE43_0.3Ca alloy extracts of different concentrations (i.e., 10%, 20%, 40%, 60%, 80%, 100%). Cell viability is expressed as a percentage in relation to the control wells (see details in the text). Significant difference (*p* < 0.05): (v)—comparing the same extract of the different tested alloys; (o)—comparing the tested extracts of the same alloy.

The results of the cytocompatibility test for a comparative evaluation of the viability profile of MG63 osteoblasts after their coincubation with extracts (obtained by immersion of the studied alloys in culture medium) of several concentrations (i.e., extract 10%, extract 20%, extract 40%, extract 60%, extract 80%, extract 100%), are plotted in Figure 9.

The colorimetric tests with MTT for cell viability assessment one day after MG63 cells were cultured with the selected extracts of the studied alloys (i.e., WE43_0.2Ca, WE43_0.3Ca) showed that, for the 10%, 20%, and 40% extracts of both alloys, no significantly different values were recorded (*p* > 0.05). In addition, the viability profile of the WE43_0.2Ca alloy was significantly different (*p* < 0.05) and higher in the case of the 60%, 80%, and 100% extracts compared to the viability attained for the same concentrations in the case of the WE43_0.3Ca alloy.

After 3 days of cell coincubation with the selected extracts (10%, 20%, 40%, 60%, 80%, and 100% concentrations), the cell viability increased for all the studied dilutions compared to cell viability after 1 day (i.e., increase in cell viability profile with increases in both the time and extract concentrations), and this finding was similar for both studied alloys (WE43_0.2Ca, WE43_0.3Ca). Furthermore, when comparing the studied alloys with each other, the cell viability at 3 days was not significantly different (*p* > 0.05) for any of the studied extracts. However, the cell viability recorded after 3 days of culture for the 80% and 100% extracts was significantly higher (*p* < 0.05) compared to the other dilutions studied, a feature recorded in the case of both studied alloys.

**Figure 10:**
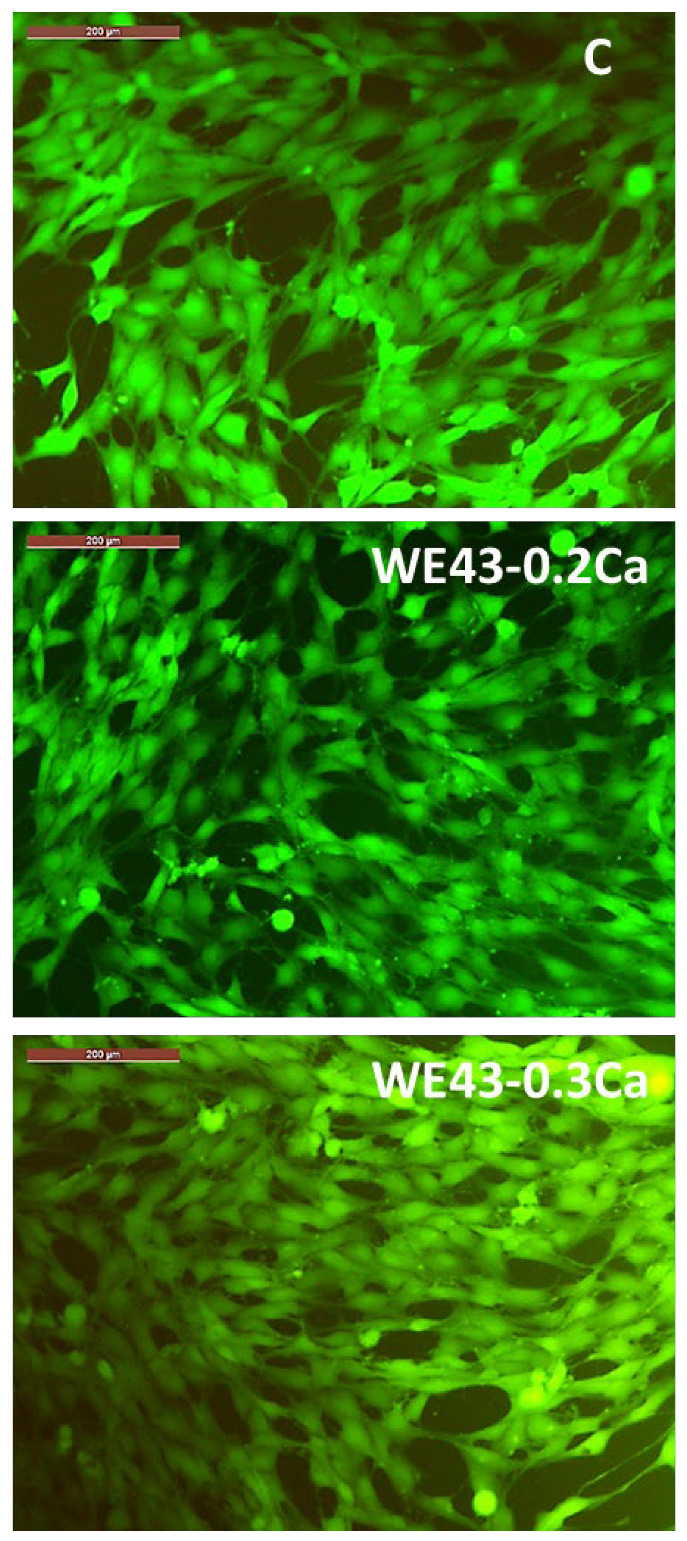
Images captured with a fluorescence microscope highlighting the morphology of MG63 osteoblasts coincubated for 3 days with 100% extract for each of the alloys studied (WE43_0.2Ca and WE43_0.3Ca) and the control well (C). Cells stained with Calcein–AM. Scale bar 200 µm.

In addition, from the fluorescence microscopy images (Figure 10), it can be observed that the MG63 cells coincubated for 3 days with 100% extracts of the studied alloys (i.e., WE43_0.2Ca and WE43_0.3Ca) have a polygonal morphology with large cytoplasmic processes of the filopodia or lamellipodia type, similar to the morphology of cells in the control well (i.e., cells cultured only with the culture medium, without the addition of extract).

The fact that the viability levels after 3 days of MG63 cell culture with the 80% and 100% extracts were higher than those after 1 day suggests a long-term favorable effect of ionic composition on stimulating cell proliferation, behavior which could be explained by adaptive physiological mechanisms to an microenvironment rich in ions with physiological roles, with calcium ions playing a well-known, fundamental role not only in the reorganization of cytoskeleton elements but also in stimulating the proliferation, differentiation, and mineralization of the extracellular matrix, as well as an implicit role in the process of osteogenesis and osteointegration [66]. In addition, Mg ions are essential to the regulation of various cellular functions, such as osteoblast cell proliferation, and the expression levels and activities of several key osteogenic markers, such as alkaline phosphate and osteocalcin [67].

In this sense, the fluorescence microscopy images (Figure 10) revealed that the cellular morphology was not affected by the coincubation of MG63 cells with an extract containing a 100% concentration of the studied alloys (i.e., WE43_0.2Ca and WE43_0.3Ca) for 3 days.

Consequently, the cell viability data evidenced the non-cytotoxic behavior [44] of the studied alloys (WE43_0.2Ca, WE43_0.3Ca), since the cell viability values were maintained in all cases at values closer to or higher than those of the control wells, and the cell morphology did not undergo changes. Accordingly, the viability values of MG63 cells cultured with 100% extract, after one day of culture, were 122.796% ± 3.452 in the case of the WE43_0.2Ca alloy and 92.401% ± 3.685 for the WE43_0.3Ca alloy, and after 3 days of culture, levels of 164.825% ± 5.561 were attained by WE43_0.2Ca and 169.060% ± 5.976 was attained by the WE43_0.3Ca alloy. In addition, our results are consistent with a recently published study on the WE43 alloy that demonstrated the favorable effect of the WE43 alloy on the behavior of major bone cells (i.e., both osteoblasts and osteoclasts) [68].

## 4. Conclusions

Following the microstructural analysis of the WE43-Zn-Ca system, a refinement of the grains and a reduction in their size was observed with the variation in Ca content from 0.2% to 0.3%, although not a significant one. Electron microscopy confirmed the absence of major defects, such as segregation, microcracks, porosity, or solidification flaws, for the WE43-Zn-XCa (x = 0.2 and 0.3) alloy system, demonstrating the efficiency of the manufacturing process and the development of an alloy with superior structural and mechanical properties.

Due to its reduced corrosion rate and the formation of a stable protective Mg(OH)_2_ layer, the WE43_0.3Ca alloy shows favorable characteristics for use as a biomaterial, providing good resistance in physiological environments and effective protection against premature degradation.

The reduction in stiffness, along with the lower coefficient of friction and an elastic modulus closer to that of human bone, qualifies the WE43_0.3Ca alloy for repeated long-term use, indicating it has higher abrasion resistance and lower localized wear compared to WE43_0.2Ca.

The results of the cell viability assay of osteoblasts from the MG63 cell line proved the non-cytotoxic behavior of the alloys studied (WE43_0.2Ca and WE43_0.3Ca), since the viability profile of cells cultured for 1 day with the 100% extract was 122.796% ± 3.452 in the case of the WE43_0.2Ca alloy and 92.401% ± 3.685 for the WE43_0.3Ca alloy, while after 3 days of culture, the profile was 164.825% ± 5.561 for WE43_0.2Ca and 169.060% ± 5.976 for the WE43_0.3Ca alloy, without affecting the cell morphology.

The studied Mg-based alloys demonstrated outstanding potential as biomaterials due to their unique combination of biocompatibility, an elastic modulus close to that of human bone, and their favorable mechanical properties. Their bioresorb ability within the biological environment, reducing the need for secondary surgical procedures to remove implants, provides a significant advantage over conventional materials, and after further carefully adjustments to their composition, surface treatments, and manufacturing techniques, the studied Mg alloys can be optimized for orthopedic, cardiovascular, and dental applications, contributing to tissue regeneration and effective integration within the human body.

## Figures and Tables

**Table 1:** Chemical composition of the WE43, WE43_0.2Ca, and WE43_0.3Ca alloys (wt).

System	Mg	Ca	Zn	Y	Zr	Nd	Dy	Gd
WE43	97	-	-	1.16	1.14	0.7	0.4	0.28
WE43_0.2Ca	93	0.284	2	2.14	0.6	0.6	0.4	0.29
WE43_0.3Ca	95	0.31	1.63	0.7	0.25	0.23	0.4	0.13

**Table 2:** Corrosion process parameters.

System	Corrosion Process Parameters					
	E_cor_ (mV)	i_cor_ (mA/cm^2^)	Rp (ohm.cm˛)	v_cor_ (mm/Y)	−β_c_ (mV/dec)	β_a_ (mV/dec)
WE43	1561	0.19	121.6	4.59	151	128
WE43_0.2Ca	−1565	0.15	341	3.46	299	126
WE43_0.3Ca	−1573	0.11	174	2.6	129	86

**Table 3:** Parameters obtained by fitting the EIS data using the equivalent circuit model R(Q(R(QR))).

Sample	R_s_ Ω·cm^2^	CPE1		R_1_ Ω·cm^2^	CPE2		R2 Ω·cm^2^
		Q_1_ Ss^n^/cm^2^	n		Q2 Ss^n^/cm^2^	n	
WE43	199	1.7 × 10^−8^	0.93	250	2.3 × 10^−4^	0.55	607
C2	202	1.67 × 10^−8^	0.96	216	5.7 × 10^−5^	0.64	1758
C3	234	1.4 × 10^−8^	0.90	592	3.33 × 10^−5^	0.69	2402

**Table 4:** Average values for stiffness, depth, Young’s modulus, and hardness for alloys WE43_0.2Ca and WE43_0.3Ca.

	We43 Alloy	WE43_0.2Ca Alloy	WE43_0.3Ca Alloy
Cof	0.17	0.16	0.14
Stiffness (N/µm)	4.7	2.8	2.9
Depth (µm)	11.2	12.6	11.8
Young’s (Gpa)	35.1 ± 0.5	19.2 ± 0.5	20.3 ± 0.5
Hardness (Gpa)	0.66	0.59	0.63

## Data Availability

The original contributions presented in the study are included in the article, further inquiries can be directed to the corresponding authors.
